# Multidentate Surfactant-Dependent Synthesis of Giant Iridium Superstructures

**DOI:** 10.1021/acs.langmuir.6c00643

**Published:** 2026-04-10

**Authors:** Ramjee Balasubramanian, Maeren E. Hill

**Affiliations:** † Department of Chemistry and Biochemistry, 6042 Old Dominion University, 4501 Elkhorn Avenue, Norfolk, Virginia 23529, United States; ‡ Department of Biological Sciences, Old Dominion University, Norfolk, Virginia 23529, United States

## Abstract

Giant superstructures of iridium, comprised of smaller spherical and slightly larger anisotropic nanoparticles, were synthesized by reducing iridium chloride in the presence of resorcinarene, a class of tetrameric macrocyclic polyphenols, with sodium borohydride in ethanol in 30 min under mild conditions. These superstructures were characterized by a range of techniques including TEM, HRTEM, EDS and other spectroscopic methods. The impact of the macrocyclic surfactant, its features and reaction conditions in dictating the formation of giant iridium superstructures was evaluated. The packing density of nanoparticles in these giant iridium superstructures could be altered qualitatively by varying the duration of the reaction, concentration, or chain length of the resorcinarene surfactant, while their overall dimensions remained in the range of 173–197 nm. Control experiments carried out in its absence and in the presence of resorcinol revealed that resorcinarene surfactant plays the role of a dispersant and further regulates the formation of near spherical aggregates. Tween 20, a nonionic surfactant, also resulted in the formation of slightly larger giant iridium superstructures with somewhat limited dispersion stability. The nature of the macrocyclic surfactant’s headgroup played a major role in the formation of these giant superstructures, as replacing resorcinarene with related pyrogallolarene only led to individually dispersed spherical and anisotropic nanoparticles though both these surfactants act as effective stabilizers for iridium nanoparticles. The available evidence suggests that the formation of these giant superstructures proceeds via the growth of sparsely populated spherical aggregates, which are formed as early as 10 min. These giant iridium superstructures showed enhanced specific activity toward oxygen evolution reaction when compared to a commercially available catalyst.

## Graphical abstract

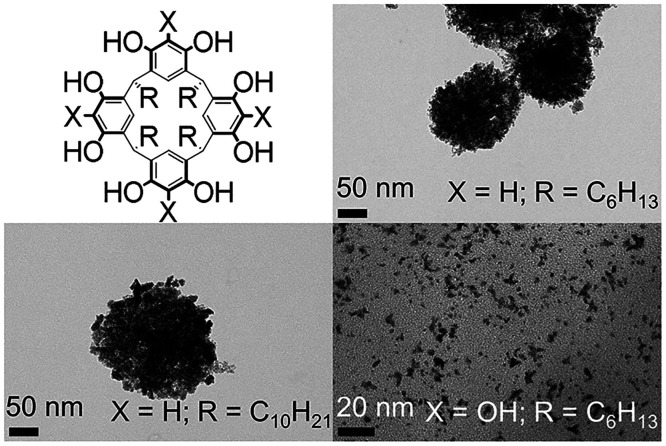

## Introduction

Iridium, one of the least abundant noble elements, has attracted a lot of attention in recent years owing to its exceptional catalytic- and electrocatalytic- activity and stability.^1–3^ Iridium nanoparticles can offer unique opportunities when compared to iridium based homogeneous catalysts.^4^ They have been applied to a variety of reactions including water splitting,^5–12^ hydrogenation,^13–15^ oxidation^16^ and site-selective deuteration^17^ among others. Surfactant ligands play a major role in dictating the dimensions and catalytic abilities of iridium nanoparticles,^18,19^ and they can also be introduced via postsynthetic approaches.^19^ Often smaller, well-dispersed roughly spherical iridium nanoparticles are formed due to the low energy barrier for homogeneous nucleation when compared to heterogeneous nucleation.^20^ Nevertheless, recent advances have enabled the synthesis of morphologically distinct iridium nanostructures such as nanoparticles,^5,9,11–13,19,21–23^ nanowires,^24^ porous nanowires,^25^ nanochains,^26^ nanotubes,^27^ nanosheets,^10,28^ mesoporous nanosheets,^29^ hollow spheres,^30^ porous structures,^31^ and nanodendrites^32^ among others. Further, iridium-based nanostructures with a plethora of additional morphologies such as nanorods, nanoframes, nanocages etc. have also been fabricated.^7,33^

Direct synthesis of 3D nanomaterials assembled from other morphologically distinct subunits have attracted a lot of attention in recent years owing to their high surface area, porosity and increased number of active sites leading to superior electron transport and enhanced stability.^7^ Iridium based 3D superstructures comprising of nanoparticles,^34,35^ nanodendrites^36^ or nanosheets^37^ have been fabricated and employed as electrocatalysts^36,37^ or in biosensing.^35^ Such structures labeled as raspberry type nanoparticles,^34^ nanopompons,^36^ superstructures^37^ or simply nanoparticles^35^ were synthesized from various precursors in the presence of surfactants such as butylamine,^34^ citric acid,^35^ cetrimonium bromide, glucose and olelylamine,^36^ and citric acid, glyoxal and poly­(vinylpyrrolidone)^37^ often at high temperatures.^35–37^ Despite these advances, newer approaches are needed to prepare 3D nanomaterials with controlled porosity, morphology and exposed crystal facets for a variety of applications.^7^

Supramolecular macrocyclic building blocks such as cyclodextrin, calixarene, cucurbituril, resorcinarene (calix[4]­resorcinarene) and their cavitands, and recently pillararene have been employed widely in the synthesis and functionalization of nanomaterials toward a number of applications.^38–41^ To the best of our knowledge, only a very limited number of reports have focused on the synthesis of iridium-based nanoparticles in the presence of multidentate surfactants and they have yielded relatively smaller nanoparticles. Katz and co-workers have employed calixarenes functionalized with phosphine ligands to stabilize atomically precise iridium (Ir_4_) clusters.^42^ Anisotropically shaped well-dispersed iridium oxide nanoparticles with an average diameter of 1.75 nm were synthesized with cyclodextrin which showed multienzyme like activity.^43^ Well-dispersed 2.0 nm sized iridium nanoparticles were obtained when iridium chloride was reduced in the presence of cucurbituril in ethanol which acted as a bifunctional catalyst for both oxygen evolution reaction and hydrogen evolution reaction.^44^ Further, cucurbituril has been used as a template for complexing iridium cations which subsequently led to the formation of 1.74 nm sized nanoparticles after pyrolysis.^45^

Resorcinarenes, a class of tetrameric macrocyclic polyphenols, and cavitands, their rigid counterparts are widely studied hosts in supramolecular chemistry.^46,47^ Both unfunctionalized^48^ and functionalized resorcinarenes,^49–52^ and resorcinarene cavitands^53^ have been employed as surfactants in the synthesis, passivation and self-assembly of metal nanoparticles.^38^ Our group has been working with functionalized, multidentate resorcinarene cavitand based surfactants for the synthesis,^54^ functionalization^55^ and phase-transfer^56,57^ of various nanomaterials toward applications in catalysis,^54^ sensing^55^ and growth of diamond films.^56^ Herein we show that simple unfunctionalized resorcinarenes **1** and **2** (Figure 1) can lead to the fabrication of giant superstructures of iridium nanoparticles. Our work shows that by varying the duration of the reaction, the concentration or the chain length of the resorcinarene surfactant, the packing density of the resulting giant superstructures could be altered. On the other hand, somewhat related macrocycle, pyrogallolarene^58^ (calix[4]­pyrogallolarene) **3** (Figure 1), yielded only well-dispersed nanoparticles.

**Figure 1:**
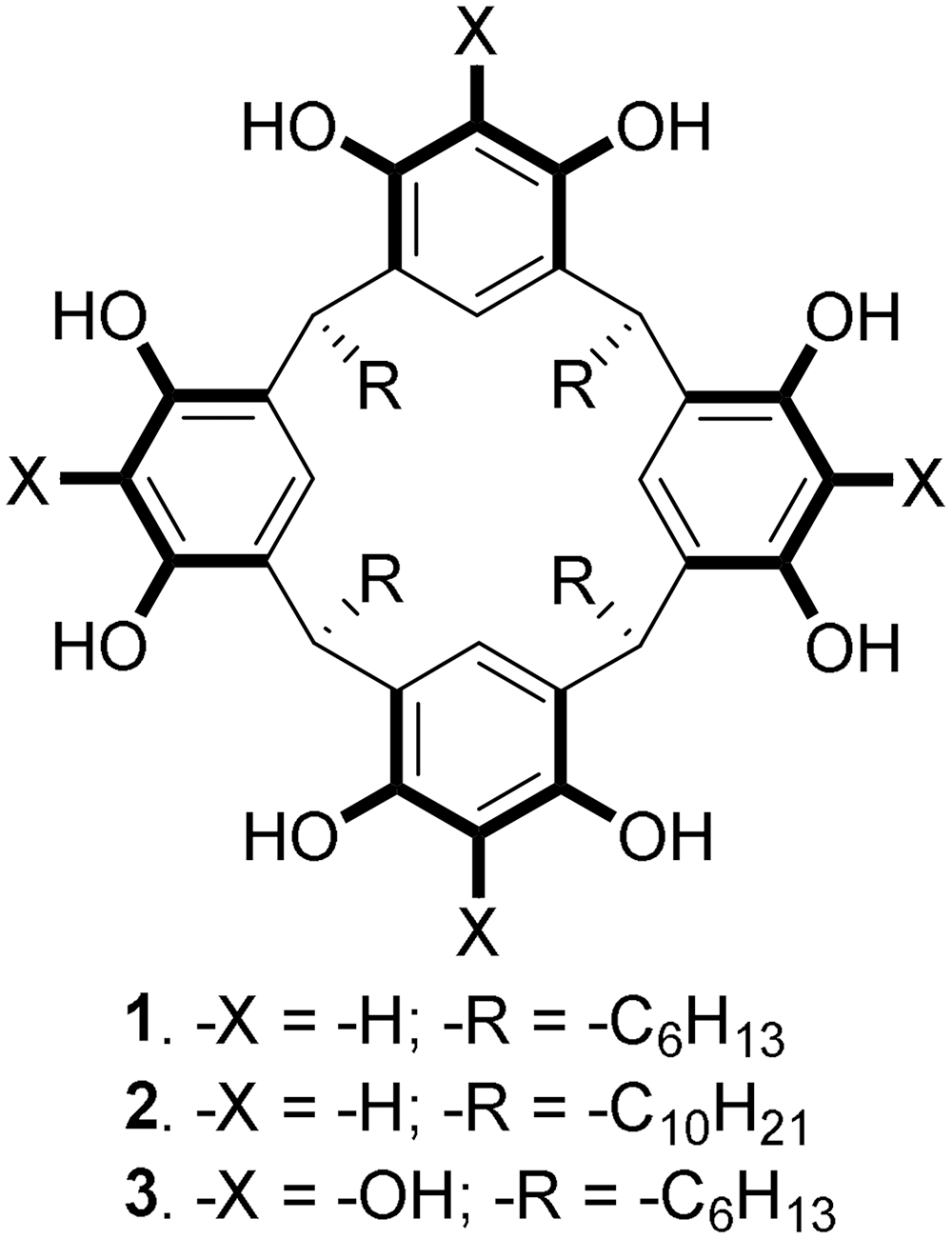
Macrocyclic surfactants.

## Experimental Section

### General Remarks

Iridium­(III) chloride hydrate (TCI America), sodium borohydride (Thermo Scientific Chemicals, 99% powder) and ethanol (95%, Decon Laboratories Inc.) were used as received. All glassware used in this study were cleaned rigorously with aqua regia (1:3 ratio of ACS grade 68–70% nitric acid and 37% hydrochloric acid) and washed with Synergy ultrapure (type 1) water (18.2 MΩcm) prior to use.

### Synthesis of Resorcinarene **1**

To a stirred solution of resorcinol (2.931 g, 26.62 mmol) and heptanal (4.3 mL, 30.76 mmol) in ethanol (17 mL) under argon atmosphere, HCl (12 N, 4.5 mL) was added dropwise at room temperature and after 5 min the reaction mixture was heated to 80 °C. After 15.5 h of heating the reaction mixture was allowed to cool to room temperature and the semisolid obtained was transferred into 150 mL of ice-cold H_2_O. After 30 min of cooling, the precipitate obtained was filtered, washed with copious amounts of hot water, and dried. The crude product obtained as a light orange powder in near quantitative yield was dissolved in methanol (18 mL per gram), boiled with activated charcoal (∼2.3 wt %), filtered, precipitated by dropwise addition of water (4.5 mL per gram) to obtain the reprecipitated title compound as a light-yellow solid in 50% yield. IR (neat, cm^–1^): 3246 (br), 2954, 2928, 2857, 1619, 1499, 1451. ^1^H NMR (DMSO-*d*_6_, 400 MHz): δ 8.85 (s, 8 H), 7.15 (s, 4 H), 6.14 (s, 4 H), 4.21 (t, 4 H, *J* = 7.8 Hz), 2.02 (m, 8 H), 1.22 (m, 8 H), 0.84 (t, 12 H, *J* = 6.8 Hz). Resorcinarene 2 was synthesized by a similar approach.

### Synthesis of Pyrogallolarene **3**

To a stirred solution of pyrogallol (3.545 g, 28.11 mmol) and heptanal (4.2 mL, 27.90 mmol) in ethanol (18 mL) under argon atmosphere, HCl (12 N, 5 mL) was added dropwise at room temperature and after 5 min the reaction mixture was heated to 85 °C. After 2 h of heating, the reaction mixture was cooled to room temperature and transferred into 150 mL of ice-cold H_2_O. After 30 min of cooling, the precipitate obtained was filtered, washed with copious amounts of water, and dried to obtain the title compound as a maroon solid (6.060 g, 98%). The crude product obtained was dissolved in ethanol (3 mL per gram), precipitated by dropwise addition of water (2 mL per gram), filtered, washed with methanol (20 mL) to obtain the reprecipitated title compound as an off-white solid in 49% yield. IR (neat, cm^–1^): 3574–3317 (br), 2951, 2928, 2854, 1610, 1476. ^1^H NMR (CDCl_3_, 400 MHz): δ 8.77 (s, 4 H), 7.46 (s, 4 H), 6.88 (s, 4 H), 6.85 (s, 4 H), 4.38 (t, 4 H, *J* = 7.6 Hz), 2.23 (m, 8 H), 1.32 (m, 8 H), 0.92 (t, 12 H, *J* = 7.0 Hz).

### Synthesis of Giant Ir Superstructures with Resorcinarene **1**

IrCl_3_·*x*H_2_O (1 mM) and resorcinarene **1** (1 mM) in ethanol were sonicated for 3 min and heated at 45 °C under argon atmosphere. After 30 min of stirring at 500 rpm, a freshly prepared suspension of NaBH_4_ (52 equiv with respect to IrCl_3_, 4.2 M) in ice-cold water was added and the reaction was continued under the same conditions as before. After 30 min, the reaction mixture was precipitated by centrifugation at 8000 rpm for 10 min at 20 °C, redispersed in an equal amount of ethanol by vigorous mixing and purified 3 more times by repeating the centrifugation-reprecipitation approach. Reactions in the presence of resorcinarene **2**, pyrogallolarene **3**, resorcinol and Tween 20, and in the absence of any surfactant were also carried out under similar conditions.

### Characterization

TEM samples were prepared by drop casting ethanol dispersions of nanoparticles on carbon film coated copper grids. TEM, EDS and SAED analyses were carried out using a JEOL JEM 2100F field emission microscope operating at 200 kV equipped with an Oxford INCAx-sight EDS detector and a Gatan SC1000 ORIUS CCD camera (11 megapixel). EDS analyses were carried out in TEM mode. Nanostructure/nanoparticle dimensions and *d* spacings were measured using ImageJ software. UV–vis spectra were recorded on a Shimadzu UV-1800 spectrophotometer. FTIR spectra were recorded on a Bruker Alpha II Platinum-ATR instrument.

### Electrochemistry

Voltammetry was performed in 0.5 M H_2_SO_4_ on a BASi-RDE2 with a three-electrode system consisting of a polished glassy carbon (3 mm) rotating disk working electrode (RDE), Ag/AgCl (3 M NaCl) reference electrode and a Pt mesh counter electrode. All potentials measured were converted to reversible hydrogen electrode (RHE) using *E*_RHE_ = *E*_Ag/AgCl_ + 0.209 + 0.059 pH. The working electrode was polished with 0.05 μ alumina, sonicated in water, followed by washing with water and isopropanol, and dried under ambient conditions. ∼6 mg of catalysts were dispersed in 250 μL of isopropanol, 500 μL of water and 7.5 μL of Nafion (5 wt %) by sonication in an ice-cold water bath for at least 20 min. Catalyst inks (3 μL) were deposited on glassy carbon electrode and air-dried for at least 45 min under ambient conditions. The cell was bubbled with Ar for at least 15 min and subsequently maintained under an Ar blanket during the experiments. The activation of catalysts was performed by cycling between 0.05 and 1.5 V vs RHE for 10 cycles at a scan rate of 50 mV/s along with rotation at 1600 rpm. Electrochemically active surface area (ECSA) was determined by using double layer capacitance obtained from static cyclic voltammetry experiments between 1.1–1.2 V vs RHE at various scan rates. *C*_dl_ was measured from the slope obtained by plotting the current density variation at 1.15 V against 10, 20, 30, 40, and 50 mV/s scan rates, and a *C*_s_ value of 0.035 mF/cm^2^ was used for estimating ECSA. The activity was measured by scanning between 0.05 and 1.55 V (vs RHE) at 5 mV/s with the RDE rotating at 1600 rpm, along with an 85% *iR* compensation via positive feedback. The background correction was carried out by averaging the anodic and the cathodic scan and the data provided are normalized by ECSA. Iridium black (99.9%, Strem) was used as a standard for comparison under identical conditions.

## Results and Discussion

Giant superstructures of iridium were synthesized in the presence of a macrocyclic surfactant **1** with sodium borohydride as the reducing agent at 45 °C for 30 min under Ar atmosphere. Excess NaBH_4_ was employed to ensure the complete reduction of IrCl_3_, as unlike other noble metals, the reduction of Ir­(III) is difficult due to its inherent stability.^59^ Immediately after the addition of the reducing agent, the reaction mixture turned from yellow to black. The resulting dispersion was purified by four rounds of centrifugation-precipitation-redispersion cycles in ethanol prior to further analyses.

TEM analysis (Figure 2a) of the typical product obtained in the presence of resorcinarene **1** revealed the formation of large near spherical nanostructures with an average dimension of 181 ± 44 nm (Figure 2b). The surface of these spherical structures was rough, and their center was darker when compared to the rim (Figure 2c,2d). Some of them appeared to be fused linearly. A closer look at some less dense areas (Figure 2e,2f) revealed that these nanostructures were mostly comprised of smaller spherical nanoparticles typically in the size range of 1.5–2 nm and some elongated particles with larger edge-to-edge dimensions in the ∼2–6 nm range. The presence of both isotropic and anisotropic nanoparticle building blocks in these structures is noteworthy as other somewhat similar raspberry structures reported in the literature were formed from near monodisperse primary nanoparticles.^34^

**Figure 2:**
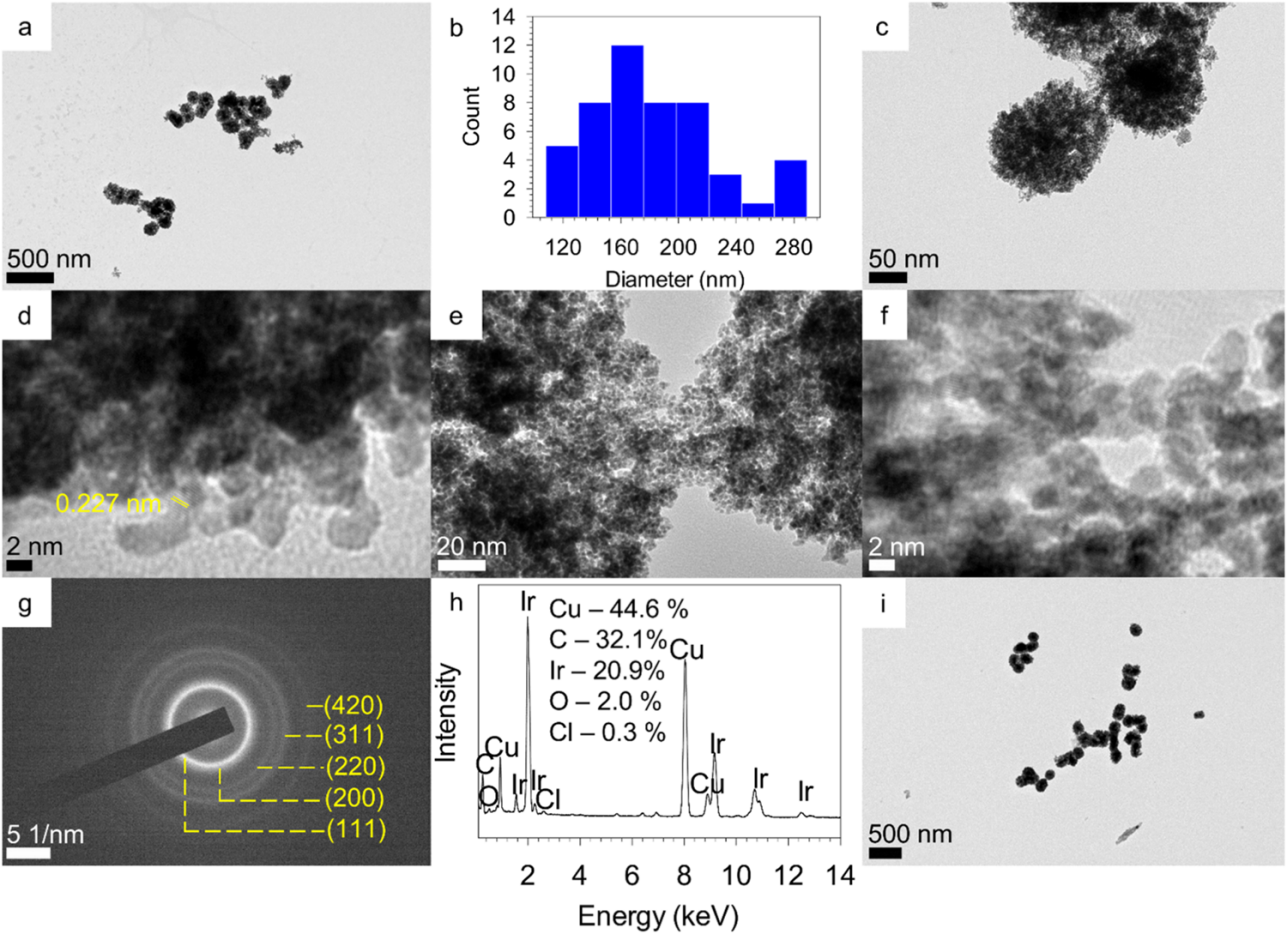
TEM (a, c, e, i), size distribution (b), HRTEM (d, f), SAED (g) and EDS (h) of giant iridium superstructures formed in the presence of resorcinarene **1** in 30 min (a–h) and 3 h (i).

HRTEM (Figure 2d,f) revealed that they are polycrystalline with a spacing of 0.227 nm corresponding to the (111) plane of Ir. The expanded image of Figure 2f is provided in Figure S1. The lattice fringes seen extensively throughout this image corresponded to the (111) plane of Ir and further showed multiple defects. The selected area electron diffraction (SAED) of these aggregates (Figure 2g) showed concentric rings indicative of their polycrystalline nature which could be indexed to (111), (200), (220), (311) and (420) planes of metallic Ir with FCC structure. The energy dispersive spectroscopy (EDS) analysis of these aggregates showed that they are composed of primarily Ir with very small amounts of O and Cl (Figure 2h), along with Cu originating from the TEM grid.^60^ The small amount (<10%) of oxygen present when compared to the iridium could be attributed to the presence of expected surface oxides resulting from their postsynthetic handling under ambient laboratory conditions and rules out the presence of IrO_2–*x*_.^34^

Some sparsely populated spherical aggregates with (multiple) dark cores (Figure S2) were also observed under these conditions. Their HRTEM analysis (Figure S2c,d) again clearly showed the extensive presence of lattice fringes corresponding to (111) plane of Ir. Outside of these spherical structures, a few isolated, crystalline, isotropic and anisotropic structures including rod-like structures with branches and V-shaped particles were also observed (Figure S3a,b). HRTEM analysis (Figure S3b) clearly showed a *d* spacing consistent with (111) plane of Ir, and further the presence of defects (red circle) and twin could be clearly seen. Consistent with the earlier estimates, the dimensions of the spherical nanoparticles were 1.5 ± 0.5 nm (Figure S3c) and the anisotropic nanoparticles had an average edge-to-edge dimension of 4.7 ± 1.7 nm (Figure S3d). The EDS analysis of these individual nanoparticles again showed the presence of Ir, C and Cu along with small amounts of oxygen (Figure S3e).

The extinction spectrum of these superstructures was distinct (Figure 3, **1**) in contrast to the exponential decay pattern usually observed for smaller, spherical iridium nanoparticles by various groups.^19,22,23^ However, theoretical calculations^61,62^ have predicted a somewhat similar pattern, especially in the visible region, for larger anisotropic structures. Further, the lack of any significant absorbance maxima around 575–580 nm confirmed the absence of IrO_2_ ^63^ or other Ir*_n_* O*_m_* ^64^ oligomers in agreement with the EDS results.

**Figure 3:**
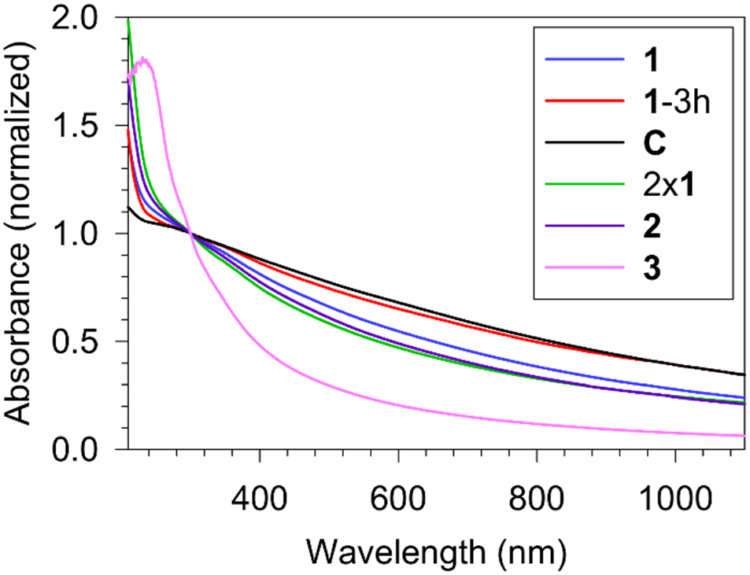
UV–vis spectra of iridium nanostructures prepared in the presence of surfactants **1**–**3** and control (C) in the absence of any surfactant in 30 min (except **1**–3 h).

The FTIR of both resorcinarene **1** and the nanoparticles prepared in its presence are shown in Figure S4. The OH stretch at 3262 cm^–1^ in the parent resorcinarene was shifted to 3340 cm^–1^ in the nanoparticles. The C–H stretches at 2954 cm^–1^, 2858 cm^–1^ and 2927 cm^–1^ in the parent resorcinarene **1** could not be clearly seen in the giant superstructures prepared due to the presence of strong OH stretch. The aromatic C=C stretch at 1618 cm^–1^ in the parent resorcinarene was shifted to 1656 cm^–1^ in the superstructures. Notably, most of the peaks in the fingerprint region of the nanoparticles were shifted when compared to the parent surfactant. Also, the bands in the functional group region were attenuated when compared to the ones in the fingerprint region. Though the specific reasons are unclear at this point, these observations are not entirely surprising as FTIR of DNA templated iridium nanoparticles also differed when compared to parent DNA.^65^

To get a better understanding of the formation of these superstructures we varied several critical parameters in the reaction. When the reaction was allowed to proceed for 3 h, very similar giant superstructures (Figures 2i and S5) with slightly larger dimensions of 197 ± 48 nm were obtained. In contrast to the 30 min reaction, some sedimentation was observed at the very end of the 3 h reaction. Sparsely populated spherical aggregates such as those in Figure S2 were absent under these conditions. Their extinction spectrum appeared almost linear except in the UV region (Figure 3, **1**–3 h). On the other hand, the giant iridium superstructures (184 ± 40 nm) formed after 1 h of reaction also contained some sparsely populated structures (Figure S6). Interestingly, giant iridium superstructures (182 ± 43 nm) were obtained even after 10 min of reaction (Figure S7). Not surprisingly, most of them were sparsely populated aggregates and clearly showed the presence of core–shell architecture.

To probe the importance of the multidentate surfactant in the formation of spherical nanoparticle aggregates, a control reaction was carried out in its absence. Within 10 min of the addition of sodium borohydride, the reaction mixture precipitated completely in the flask despite vigorous stirring. The suspension required relatively longer periods of sonication unlike the iridium superstructures synthesized in the presence of resorcinarene which could be redispersed readily by vigorous agitation or brief sonication. Their TEM analysis revealed that iridium nanoparticle aggregates with a range of sizes and shapes including some spherical aggregates (Figure S8a–c) were formed. This is not entirely surprising as the reduction of IrCl_3_ at a higher concentration in aqueous medium with sodium borohydride has resulted in the formation of iridium foams.^63^ A closer look revealed that most of these particles were crystalline and anisotropic in nature. As before, the EDS analysis (Figure S8d) of these particles revealed that they primarily contained Ir along with very small amounts of O and Cl. Consistent with our other observations, the extinction spectrum of these nanoparticles (Figure 3, C) showed an almost straight line like feature except at the very short wavelengths confirming the presence of larger, densely packed nanoparticle aggregates. This control experiment clearly indicated that resorcinarene **1** played the role of a dispersant and further regulated the formation of spherical aggregates observed earlier.

A control reaction carried out in the presence of 4 equiv of resorcinol instead of resorcinarene surfactant under otherwise identical conditions precipitated completely during the course of the reaction. Their TEM analysis primarily showed the presence of extended aggregates and other relatively smaller randomly shaped aggregated particles of various sizes, and additionally more dense and sparsely populated regions (Figure S9). This experiment clearly revealed that the resorcinarene surfactant with its hydrophobic alkyl chains is indeed required for the formation of giant iridium superstructures, and resorcinol alone will not suffice.

When the concentration of resorcinarene **1** was doubled, very similar giant iridium superstructures (Figure S10) were obtained with many appearing to be fused linearly. A closer look revealed that their dimensions were slightly smaller (176 ± 30 nm) and they were not as densely populated. Their extinction spectrum also appeared slightly more exponential (Figure 3, 2x**1**). The FTIR spectrum of these particles clearly showed the presence of hydroxyl groups and some of the bands in the fingerprint region appear shifted as before.

Remarkably when resorcinarene **1** was replaced in the above synthesis with resorcinarene **2** with longer chains, very similar giant iridium superstructures (Figures 4 and S11) were obtained albeit slightly smaller with a dimension of 173 ± 44 nm (Figure S11a). Some less dense aggregates were also observed (red circles, Figure S11b) under the same conditions, which did not have the darker cores seen in Figure S2. Interestingly, some aggregates which appeared dark at lower magnifications (Figure S11c, blue circle) were less dense at higher magnification (Figure S11d). As before, these aggregates are formed by the aggregation of both isotropic and anisotropic nanoparticles (Figure S11d).

**Figure 4:**
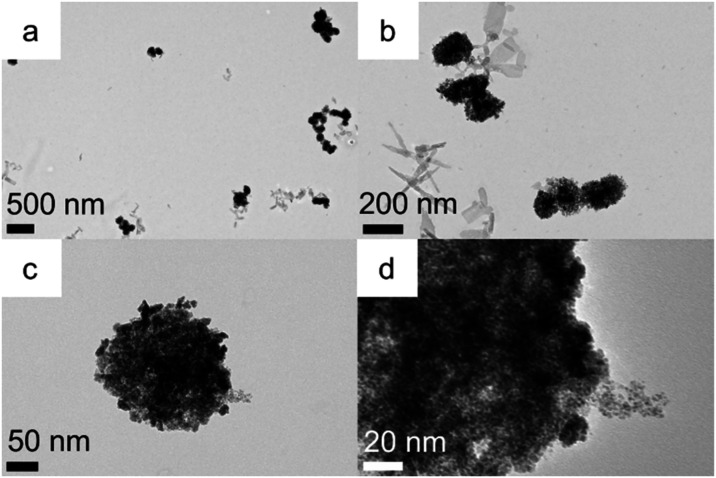
TEM (a–d) of giant iridium superstructures prepared in the presence of resorcinarene **2** in 30 min.

Having established the importance and impact of resorcinarene surfactant in the formation of giant iridium superstructures, we probed the role of the headgroup by replacing it with related pyrogallolarene **3**. Its TEM analysis (Figure 5a) revealed the presence of spherical nanoparticles with a dimension of ∼2.0 ± 0.5 nm and anisotropic nanoparticles which were either slightly elongated or had a V-shaped feature with longer dimensions ∼5.3 ± 0.5 nm. No giant superstructures were observed under these conditions. HRTEM (Figure 5b) clearly revealed the presence of crystalline planes with 0.227 nm spacing consistent with the presence of (111) plane of Ir. Their EDS analysis (Figure 5c) mainly showed the presence of Ir, C and Cu originating from the TEM grid,^60^ along with O and Na. In contrast to those synthesized in the presence of resorcinarenes **1** and **2**, the UV–vis spectrum of the iridium nanoparticles prepared in the presence of pyrogallolarene **3** showed a featureless exponential decay pattern (Figure 3, **3**) similar to that observed by several other groups.^19,22,23^ Figure S12 shows the FTIR analysis of nanoparticles synthesized in the presence of pyrogallolarene and the surfactant. Notably the somewhat sharp OH stretches appearing at 3573 cm^–1^, 3409 cm^–1^ and 3316 cm^–1^ presumably resulting from the intramolecular hydrogen bonding seen in the parent pyrogallolarene were missing in the nanoparticles and a broad stretch replaced the same ∼3350 cm^–1^. The C–H stretches at 2951 cm^–1^, 2925 cm^–1^ and 2854 cm^–1^ in pyrogallolarene **3** were slightly shifted to 2955 cm^–1^, 2928 cm^–1^ and 2858 cm^–1^ respectively. The aromatic C=C stretch at 1609 cm^–1^ in the parent pyrogallolarene were again shifted slightly to 1631 cm^–1^ in the nanoparticles.

**Figure 5:**
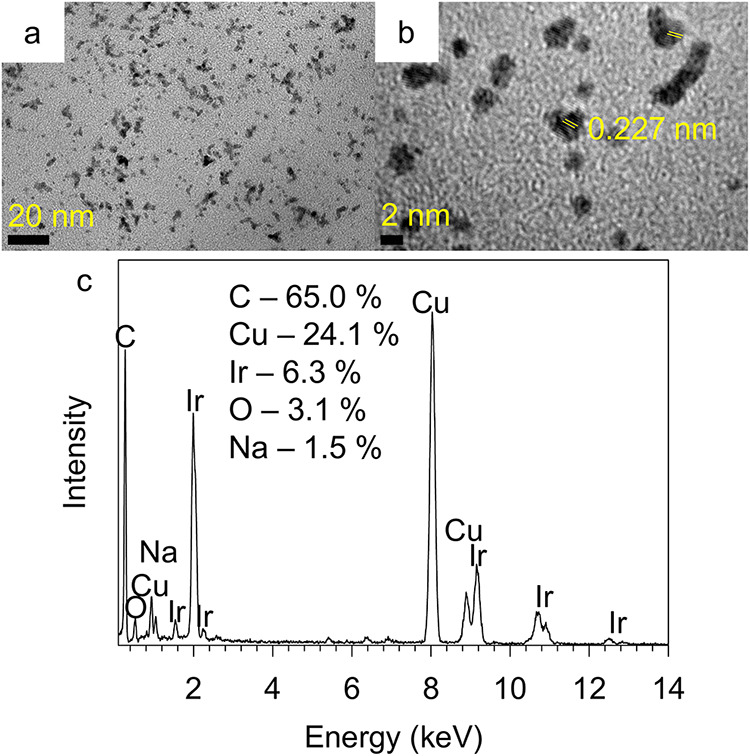
TEM (a), HRTEM (b) and EDS (c) analysis of iridium nanoparticles prepared in the presence of pyrogallolarene **3** in 30 min.

Our experiments demonstrate that resorcinarene surfactants play a critical role in the formation of these giant superstructures as only randomly shaped larger assemblies are obtained in their absence. Qualitatively, a higher proportion of spherical nanoparticles were observed in the aggregates formed when compared to anisotropic nanoparticles in the presence of resorcinarene surfactants (Figures 2, 4, S2, S3 and S11) than in their absence (Figure S8) under identical conditions and further this proportion could be increased with resorcinarene concentration (Figure S10). The higher proportion of spherical nanoparticles formed in the presence of resorcinarene **1**, resorcinarene **2** and pyrogallolarene **3** could be attributed to these surfactants’ ability to act as effective stabilizers. Additionally, the density of packing in these superstructures varied with the resorcinarene concentration (compare Figure 2 with Figure S10) or the chain length of the resorcinarene surfactant (compare Figures 2 and S2 with Figures 4 and S11). All these data suggest that these superstructures are formed by the interdigitation of hydrophobic chains of resorcinarene surfactants present on these nanoparticles. Also, the lack of formation of giant iridium superstructures in the presence of resorcinol further supported this possibility. While resorcinarene surfactant led to the formation of giant superstructures, under identical conditions, somewhat related pyrogallolarene only led to the formation of individually dispersed nanoparticles. This is not entirely surprising as tannic acid containing multiple pyrogallol units has also been reported to yield smaller, well-dispersed iridium nanoparticles with ∼3.5 nm dimensions under comparable conditions.^66^ However, the authors noted that aggregates of iridium nanoclusters were obtained when excess sodium borohydride was employed. Given this it is possible that perhaps the reaction conditions need to be optimized for the formation of giant superstructures with pyrogallolarene surfactant. The interaction of resorcinarene and pyrogallolarene surfactants with iridium nanoparticles could be different as both iridium precursors^66^ and iridium oxide nanoparticles^67^ have been documented to bind strongly with the catechol moieties present in pyrogallolarene. Resorcinarenes with hexyl chains are known to interdigitate both in solid state^68^ and in solution^55^ even when present on a self-assembled capsule or on nanoparticles, respectively. While the side chains on pyrogallolarene can also interdigitate perhaps the mode of interdigitation could be different. The specific reason for the lack of formation of giant superstructures in the presence of pyrogallolarene is unclear at this point.

The formation of aggregates under certain conditions in the presence of tannic acid^66^ is consistent with our observation of aggregates in the presence of resorcinol as they both lack hydrophobic alkyl chains. To probe the importance of both multidentate interaction and the presence of hydrophobic chain, we carried out a control experiment in the presence of Tween 20, a nonionic surfactant containing three hydroxyl groups and a laurate chain. Though this reaction also precipitated during the course of the reaction, TEM analysis (Figure 6) clearly revealed that it did lead to the formation of giant superstructures with a dimension of 212 ± 41 nm. Similar to our earlier observations, some sparsely populated giant superstructures were also observed under these reaction conditions, and they clearly showed the presence of core–shell architectures (Figure 6c,6d).

**Figure 6:**
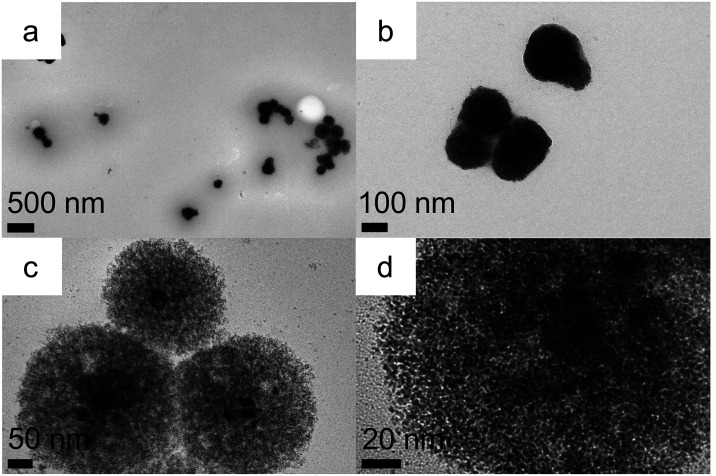
TEM (a–d) of giant iridium superstructures formed in the presence of Tween 20.

Based on these experiments, it appears that the isotropic spherical (1.5 ± 0.5 nm) and anisotropic structures (4.7 ± 1.7 nm) formed initially (Figure S3) in the presence of resorcinarene **1** assemble to form sparsely populated superstructures (Figure S2). The dominant presence of sparsely populated superstructures after 10 min of reaction (Figure S7) further supports this possibility. This appears to be a general pathway as resorcinarene **2** also led to similar structures albeit with a lower density which could be attributed to the presence of longer hydrophobic chains on resorcinarene **2** (Figure S11). Further Tween 20 too confirmed this observation (Figure 6c). Additionally, these sparsely populated superstructures with darker cores (Figures S2a,,b and S7) suggest that the mechanism of formation of well-defined giant iridium superstructures proceeds through them as they were absent when the reaction was allowed to continue for 3 h. Akin to the observations of Huang and co-workers^37^ who demonstrated the assembly and growth of nanosheets into 3D superstructures, these sparsely populated structures (Figures S2 and S7) undergo further growth to ultimately form giant iridium superstructures of varying porosities. This possibility is supported by the presence of extended lattice (Figure S2c,d) and the presence of structural defects including twin defects from individual anisotropic structures (Figure S3b) all the way to nanoparticle aggregates (Figures 2d,f and S1) suggesting that oriented attachment mechanism^35,69^ may be operational here.

Given the well-known ability of iridium nanostructures in mediating oxygen evolution reaction (OER), the utility of giant iridium superstructures was tested (Figure 7). Preliminary evaluation of the electrocatalytic activity in 0.5 M H_2_SO_4_ revealed that they showed higher specific activity toward OER when compared to commercially available Ir black. This is notable as these catalysts still contain the surfactant, which can potentially fill and block the nanopores.

**Figure 7:**
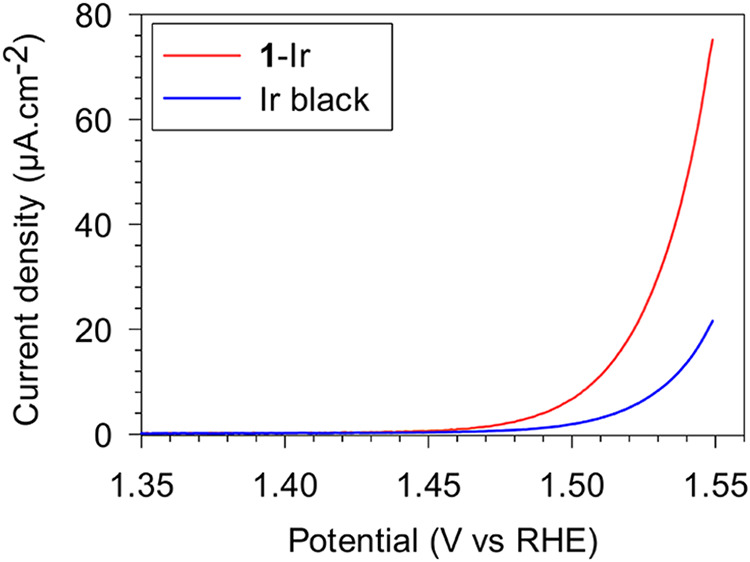
Electrochemical performance of giant iridium superstructures (**1**-Ir) and iridium black toward oxygen evolution in 0.5 M H_2_SO_4_.

## Conclusion

We have shown that relatively monodisperse giant iridium superstructures with dimensions ranging from ∼173 to 212 nm can be formed by the reduction of iridium chloride in the presence of resorcinarene and Tween 20 surfactants under mild conditions. These superstructures are comprised of spherical and other anisotropic structures including rod-like structures with branches and V-shaped particles. Our experiments have unambiguously demonstrated the crucial role played by the resorcinarene or Tween 20 surfactant as only randomly shaped assemblies of a wide range of sizes are obtained in their absence or when replaced with resorcinol. Qualitatively, the density of nanoparticle packing in these giant iridium superstructures depended on the concentration of the surfactant, the chain length of its hydrophobic tail and the duration of the reaction. Notably, the headgroup of the resorcinarene surfactant played a critical role in the formation of these giant superstructures as replacing it with related pyrogallolarene yielded only iridium nanoparticles and not superstructures. However, both resorcinarene and pyrogallolarene act as excellent surface passivating agents for iridium nanoparticles. Some sparsely populated aggregates were also observed alongside giant superstructures when the reaction was carried out for shorter durations, though they were dominant at the start of the reaction. When the reaction duration was extended, such sparsely populated structures were absent suggesting that they grow into giant superstructures. Preliminary studies showed enhanced specific activity of giant iridium superstructures in the oxygen evolution reaction when compared to a commercially available catalyst. Currently more detailed electrocatalytic activity and stability studies are underway in our laboratory and will be reported elsewhere in due course.

## Supplementary Material



## Notes

TEM, HRTEM, EDS, size distribution and FTIR of giant iridium superstructures and iridium nanoparticles (PDF)
