# Supplementary material for: Multidentate Surfactant-Dependent Synthesis of Giant Iridium Superstructures
Source: Langmuir. 2026 Apr 10;42(15):10642–50. doi: 10.1021/acs.langmuir.6c00643 (PMC13104180; doi:10.1021/acs.langmuir.6c00643)
Supplement: Supplementary file 1 [file la6c00643_si_001.pdf]

Supporting Information for

Multidentate Surfactant Dependent Synthesis of Giant Iridium Superstructures

*Ramjee Balasubramanian**,† *and Maeren E. Hill*‖

†Department of Chemistry and Biochemistry, Old Dominion University, Norfolk, Virginia 23529, United States.

‖Department of Biological Sciences, Old Dominion University, Norfolk, Virginia 23529, United States.

*Email: bramjee@odu.edu

List of Contents

1. HRTEM image of giant iridium superstructure……………………………………....S-3
2. TEM and HRTEM of sparsely populated iridium superstructures………………..…S-4
3. Analysis of individual nanoparticles formed outside the superstructures……….......S-5
4. FTIR of giant iridium superstructures………………………………………………...S-6
5. TEM analysis of giant iridium superstructures formed after 3 h….………………….S-7
6. TEM analysis of giant iridium superstructures formed after 1 h….………………….S-8

7. TEM analysis of giant iridium superstructures formed after 10 min……..…………..S-9

8. Analysis of iridium nanoparticles synthesized without any surfactant……………...S-10

9. TEM analysis of iridium nanoparticles synthesized with resorcinol………..……...S-11

10. Analysis of giant iridium superstructures……………………………….……..S-12

11. TEM analysis of less dense giant iridium superstructures…………………………..S-13

12. FTIR analysis of iridium nanoparticles……………………………………………...S-14

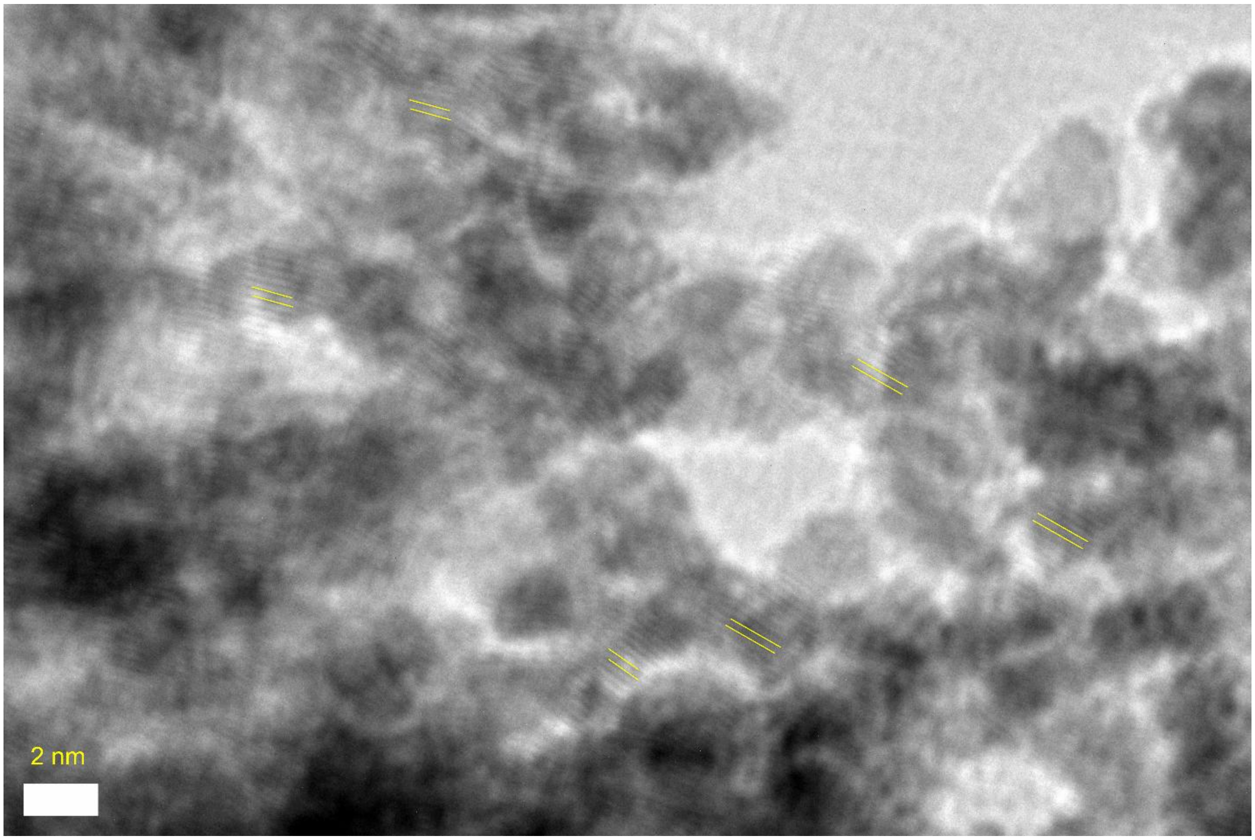

Figure S1. HRTEM image of giant iridium superstructure with d spacings (0.227 nm) corresponding to (111) planes of Ir.

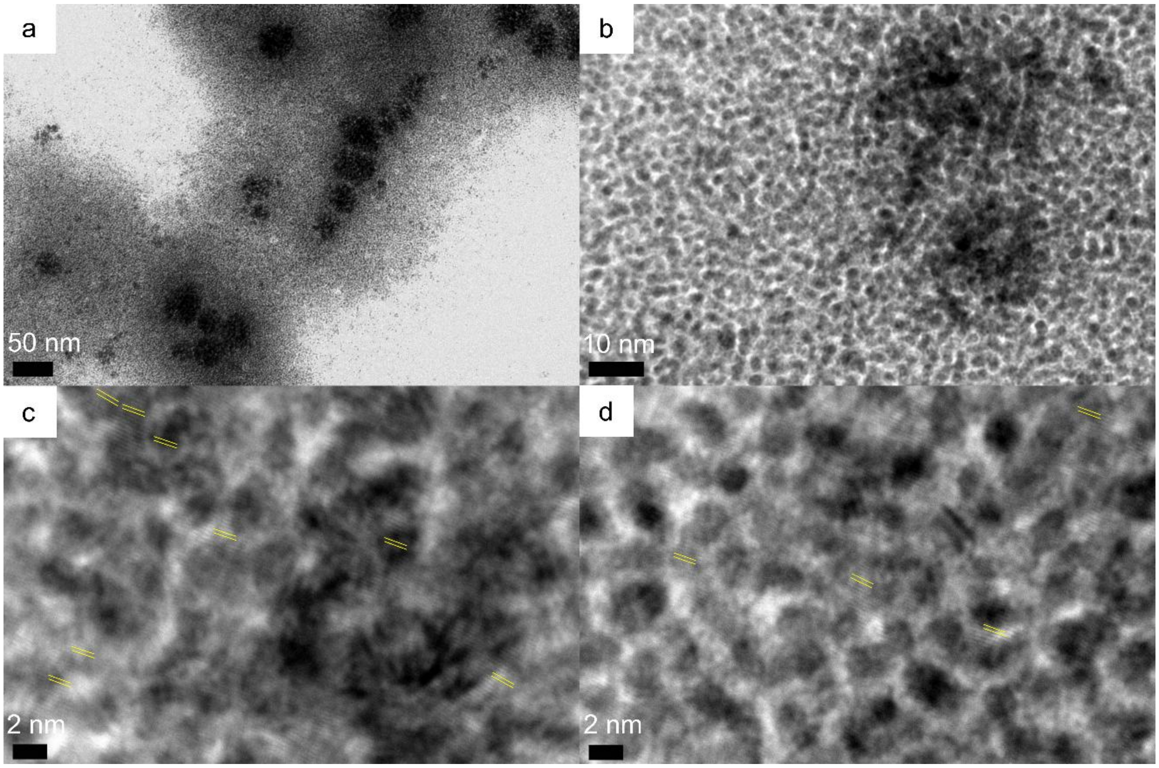

Figure S2. TEM (a, b) and HRTEM (c, d) of sparsely populated iridium superstructures formed in the presence of resorcinarene **1** in 30 min.

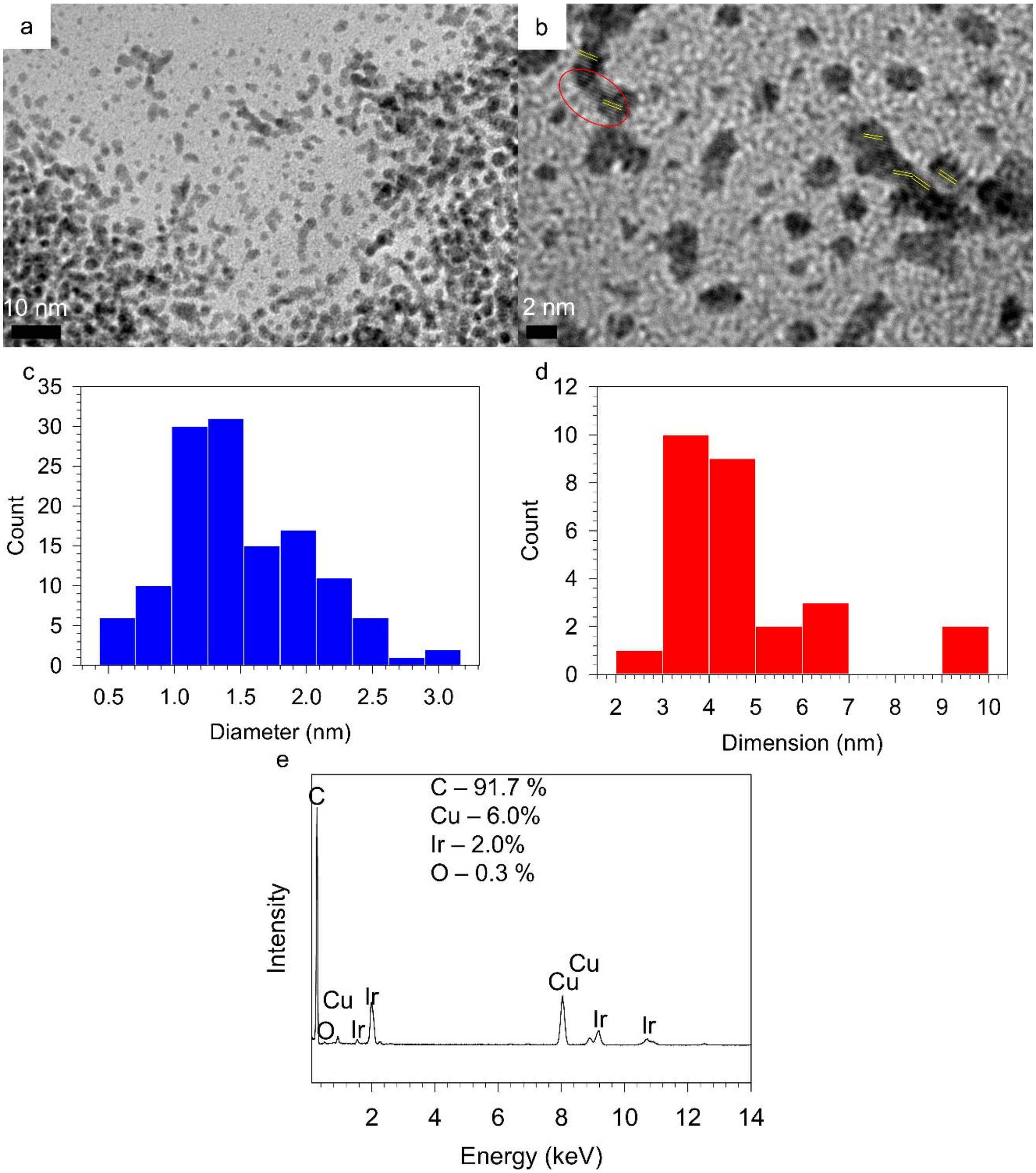

Figure S3. TEM (a), HRTEM (b), size distribution of spherical (c) and anisotropic (d) nanoparticles, and EDS (e) analysis of individual nanoparticles formed outside the superstructures in the presence of resorcinarene **1** in 30 min.

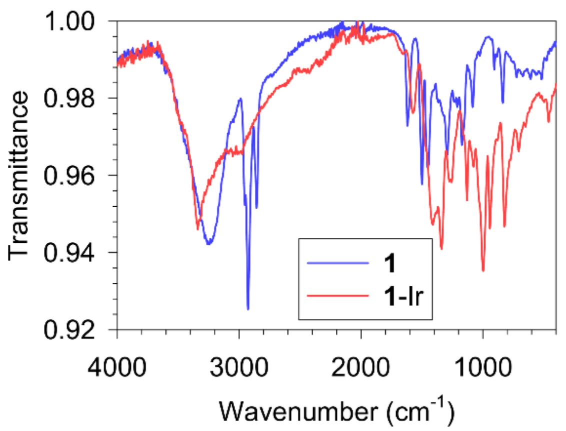

Figure S4. FTIR of giant iridium superstructures prepared in the presence of resorcinarene **1** (1–Ir) in 30 min and the parent resorcinarene **1**.

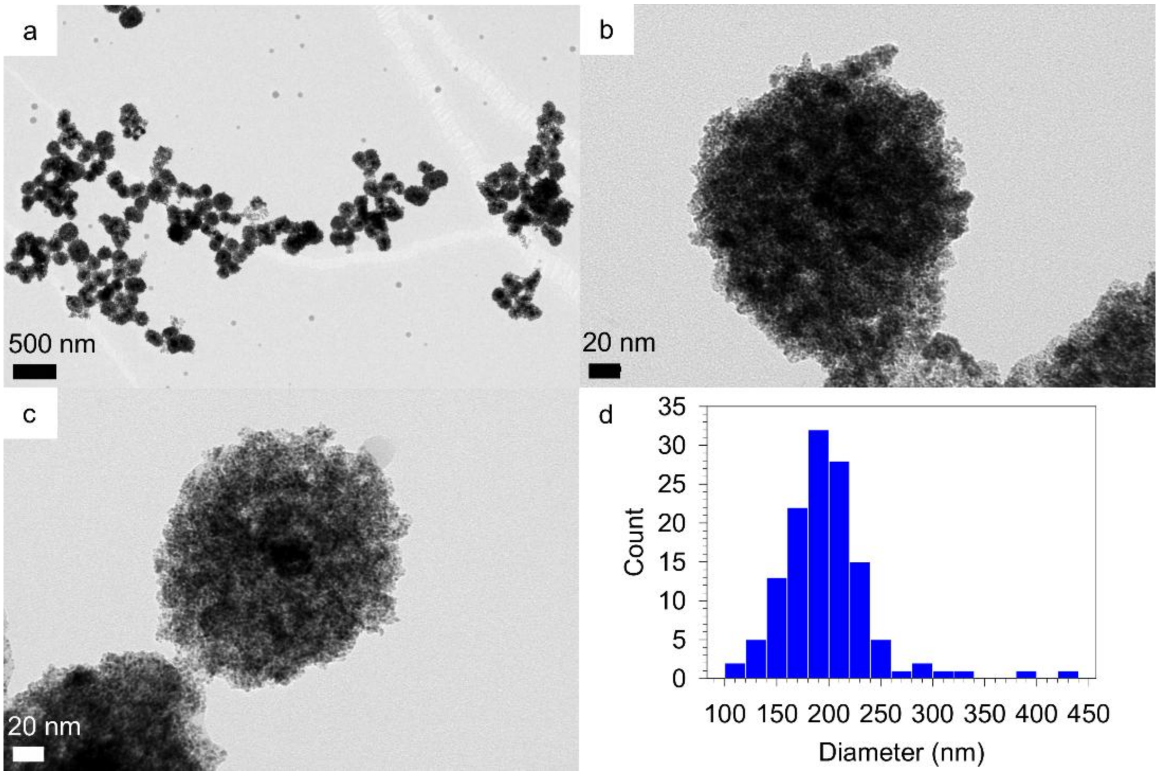

Figure S5. TEM (a – c) and size distribution (d) of giant iridium superstructures formed with resorcinarene **1** after 3 h.

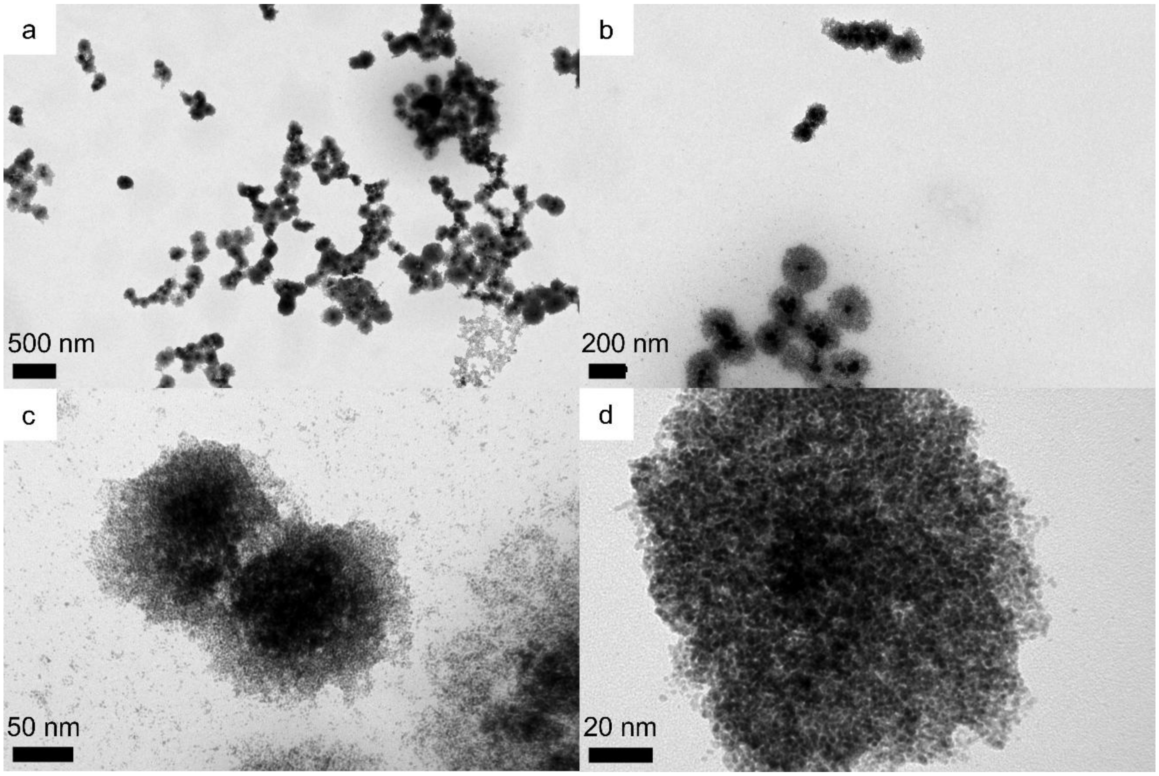

Figure S6. TEM of giant iridium superstructures formed with resorcinarene **1** after 1 h.

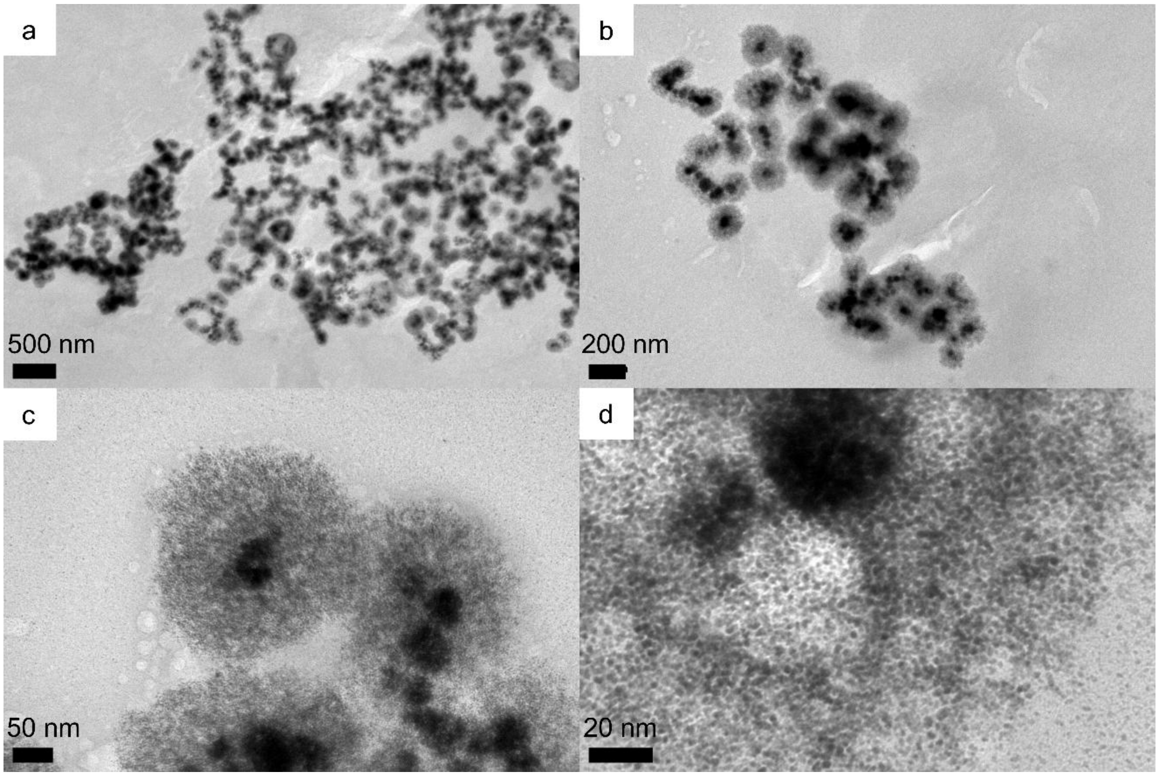

Figure S7. TEM of giant iridium superstructures formed with resorcinarene **1** after 10 min.

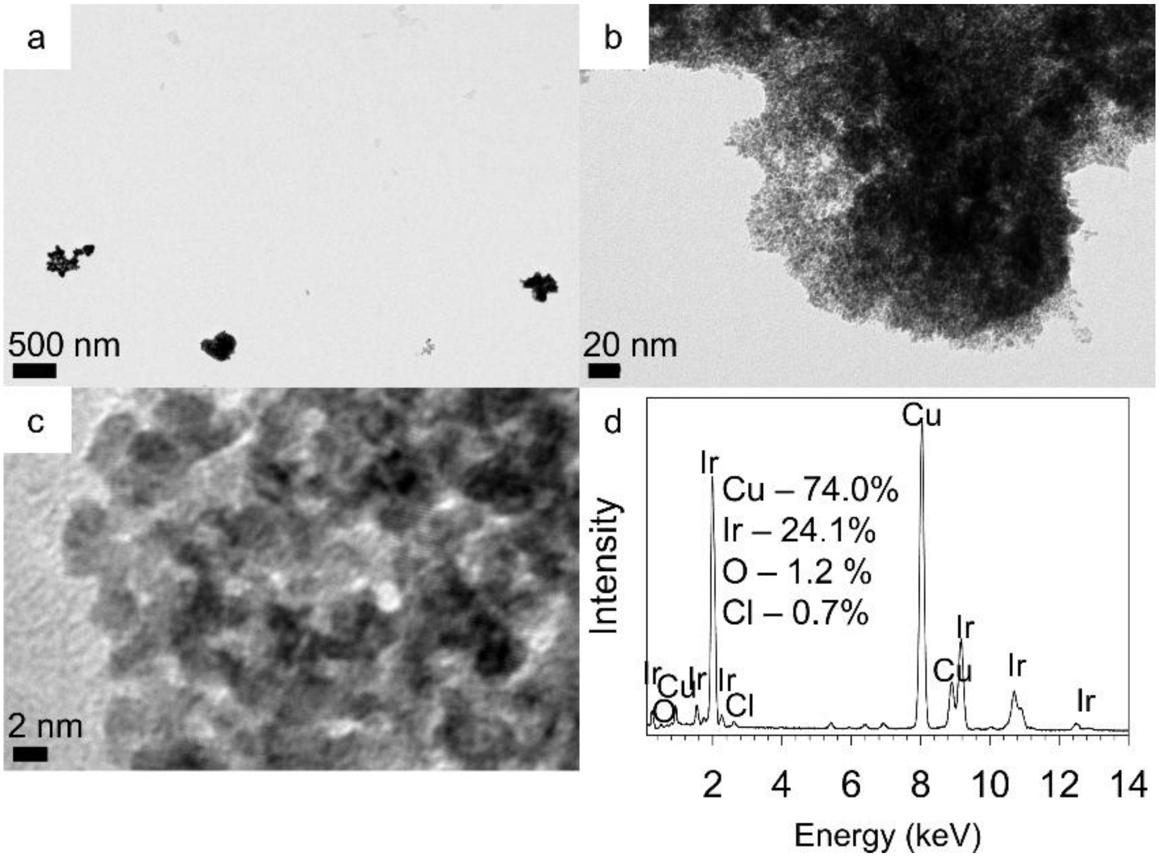

Figure S8. TEM (a – b), HRTEM (c) and EDS (d) of iridium nanoparticles synthesized without any surfactant in 30 min.

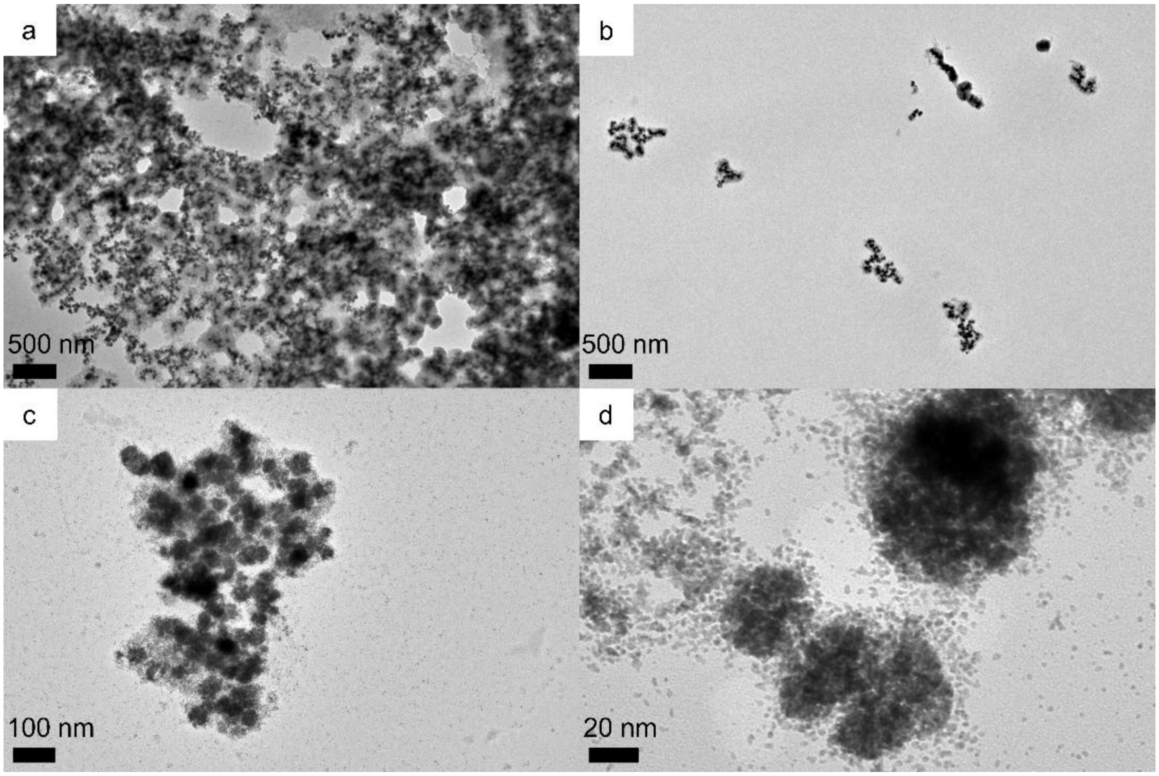

Figure S9. TEM of iridium nanoparticles synthesized in the presence of resorcinol in 30 min.

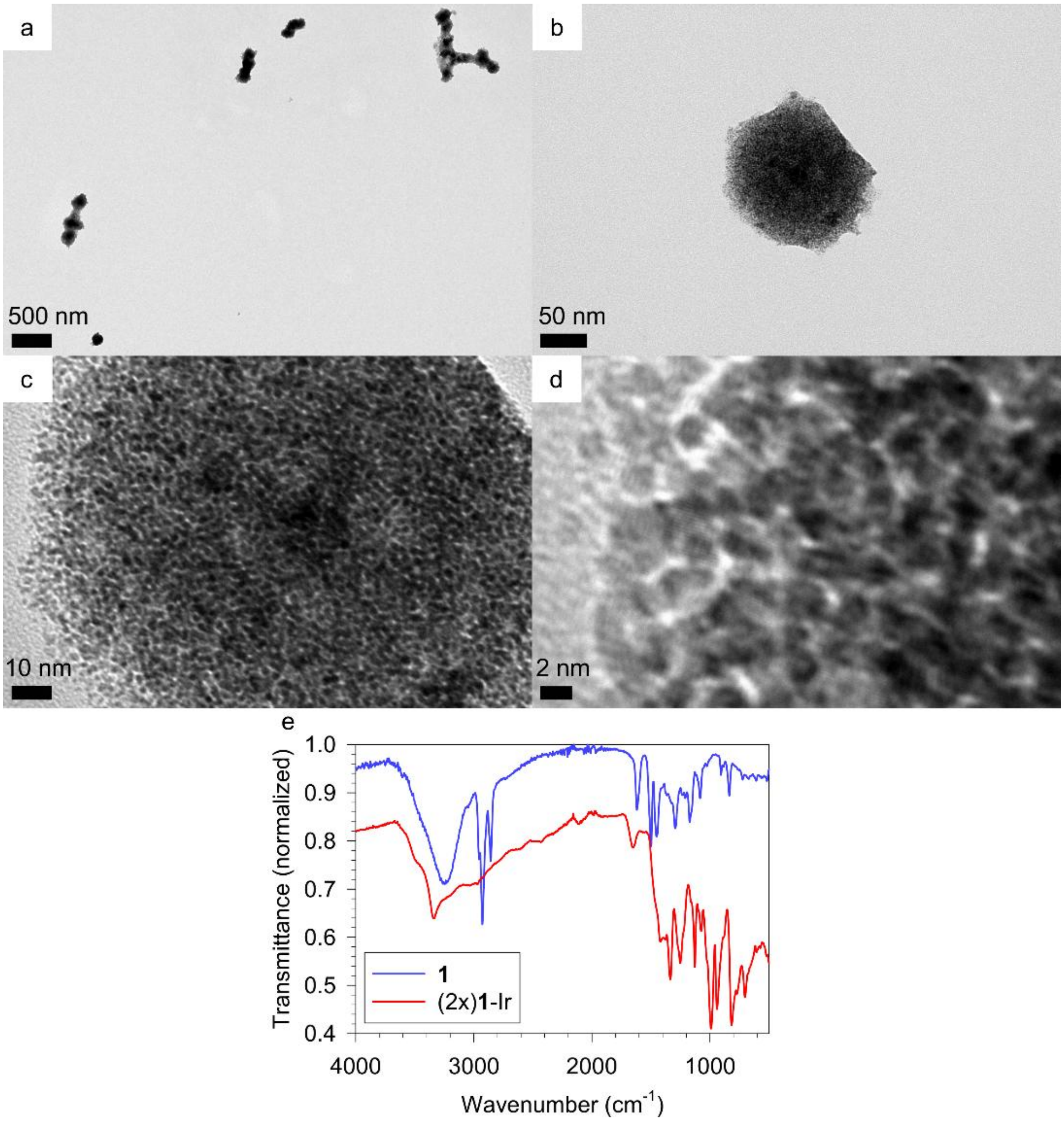

Figure S10. TEM (a – d) and FTIR (e) analysis of giant iridium superstructures ((2x)**1**–Ir) formed in the presence of 2x resorcinarene **1**.

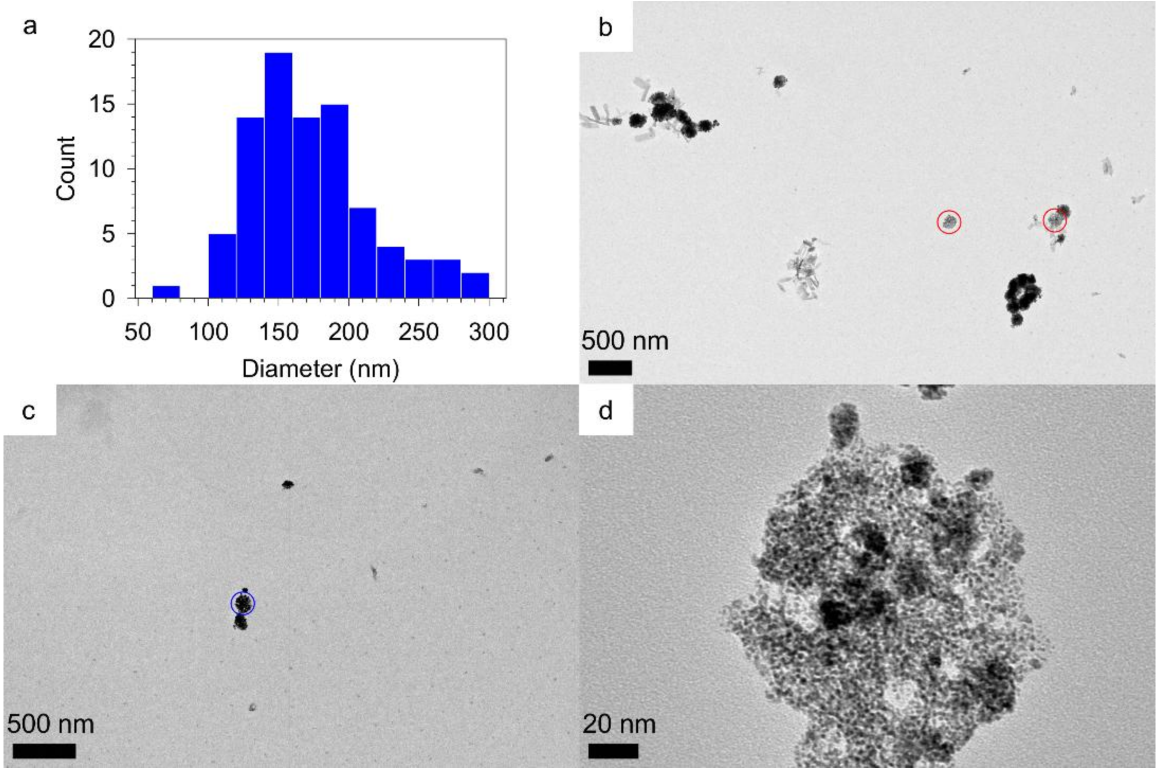

Figure S11. Size distribution (a) and TEM (b – d) of giant iridium superstructures formed in the presence of resorcinarene **2** in 30 min. Less dense structures are indicated by red circles (in S11b). Even those which appear dark at lower magnification (blue circle in S11c) were less dense (S11d) at higher magnification.

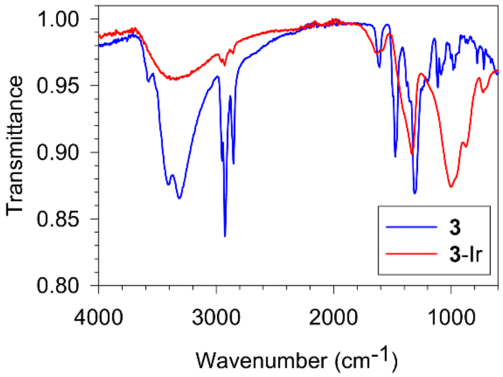

Figure S12. FTIR analysis of iridium nanoparticles prepared in the presence of pyrogallolarene **3** (**3**–Ir) in 30 min and the parent pyrogallolarene **3**.
